# Supplementary material for: AhR Activation Ameliorates Intestinal Barrier Damage in Immunostressed Piglets by Regulating Intestinal Flora and Its Metabolism
Source: Animals (Basel). 2024 Mar 4;14(5):794. doi: 10.3390/ani14050794 (PMC10931068; doi:10.3390/ani14050794)
Supplement: Supplementary file 1 [file animals-14-00794-s001.zip › animals-2839360-supplementary.pdf]

animals MDPI

Supplementary Materials

AhR Activation Ameliorates Intestinal Barrier Damage in Immunostressed Piglets by Regulating Intestinal Flora and Its Metabolism

Xiaomei Wu, Yalei Zhang, Mengyao Ji, Wen Yang, Tanjie Deng, Guanyu Hou, Liguang Shi and Wenjuan Xun

Table S1. Ingredients and nutrient levels of the basal diet (%, as-fed basis).

Ingredients	Content	Nutritional level[2]	Content
Corn	55.70	Digestible energy, MJ/kg	14.57
Soybean meal	18.80	Crude protein	20.22
Puffed soybean	8.50	Calcium	0.79
Fish meal	5.50	Total phosphorus	0.66
Whey powder	4.50	Lysine	1.52
Soybean oil	1.90	Methionine	0.50
Sucrose	1.88	Threonine	0.86
CaHPO4	0.70	Tryptophan	0.29
Limestone	0.60		
L-lysine	0.33		
DL-methionine	0.14		
L-threonine	0.05		
L-tryptophan	0.05		
NaCl	0.35		
Premix[1]	1.00		
Total	100.00		

[1] The premix provides per kilogram of feeding: VA 9 000 IU, VD3 1 200 IU, VE 65 IU, VK3 2.5 mg, VB_1 2.5 mg, VB_2 6.0 mg, VB_{12} 0.03 mg, niacin 25 mg, pantothenic acid 22 mg, biotin 0.2 mg, folic

acid 1.5 mg, copper 80 mg, iron 120 mg, manganese 45 mg, zinc 120 mg, Selenium 0.3 mg, iodine 0.3 mg.

[2] All nutrient levels were measured values except for digestible energy.

Table S2. The primers employed in real-time quantitative PCR were utilized.

Gene	Sequences	Product size	Accession numbers
ZO-1	Forward:5′GAGGATGGTCACACCGTGGT3′	169	XM003353439.1
	Reverse:5′GGAGGATGCTGTTGTCTCGG3′		
Occludin	Forward:5′ATGCTTTCTCAGCCAGCGTA3′	176	NM_001163647.1
	Reverse:5′AAGGTTCCATAGCCTCGGTC3′		
Claudin1	Forward:5′GGACTAATAGCCATCTTTGT3′	88	NM_001161635.1
	Reverse:5′CAGCCATCCGCATCTTCT3′		
IL-1β	Forward:5′ACCTGGACCTTGGTTCTC3′	124	NM_214055.1
	Reverse:5′GGATTCTTCATCGGCTTC3′		
TNF-α	Forward:5′ACGCTCTTCTGCCTACTGC3′	162	NM_214022.1
	Reverse:5′TCCCTCGGCTTTGACATT3′		
IL-10	Forward:5′CACTGCTCTATTGCCTGATCTTCC3′	136	NM_214041.1
	Reverse:5′AAACTCTTCACTGGGCCGAAG3′		
IL-6	Forward: 5′TTCAGTCCAGTCGCCTTCT3′	91	NM_214399.1
	Reverse:5′GTGGCATCACCTTTGGCATCTTCTT3′		
GAPDH	Forward:5′GAAGGTCGGAGTGAACGGAT3′	149	AF017079
	Reverse:5′CATGGGTAGAATCATACTGGAACA3′		

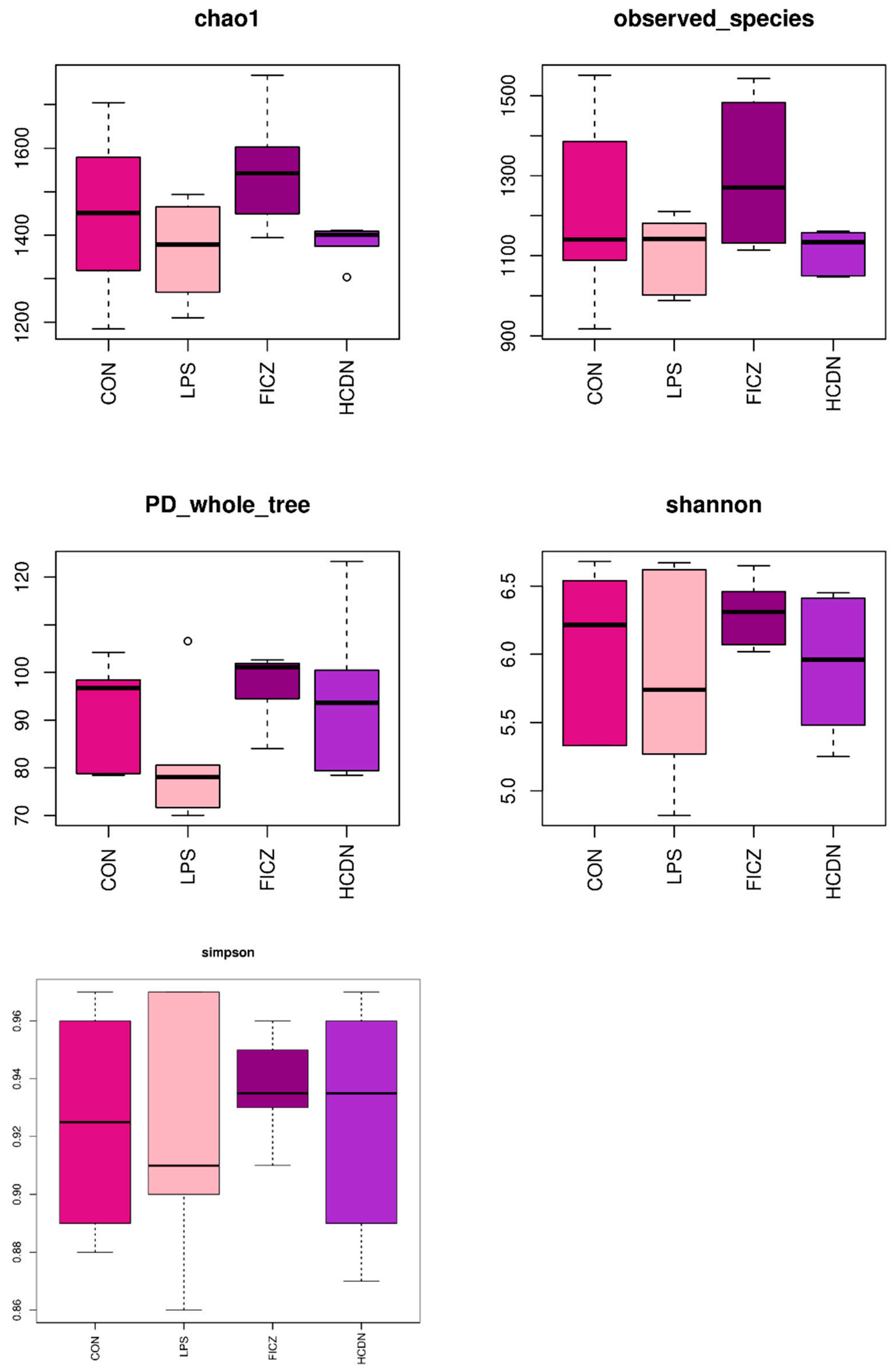

Figure S1. alpha diversity index
